# Challenges and Opportunities in Achieving Asthma Remission

**DOI:** 10.3390/jcm14082835

**Published:** 2025-04-20

**Authors:** Elena Cojocaru, Raluca Ioana Arcana, Steluta Radu, Antigona Carmen Trofor, Cristian Cojocaru

**Affiliations:** 1 Morpho-Functional Sciences II Department, Faculty of Medicine, “Grigore T. Popa” University of Medicine and Pharmacy, 700115 Iasi, Romania; elena.cojocaruu@umfiasi.ro; 2 Medical III Department, Faculty of Medicine, “Grigore T. Popa” University of Medicine and Pharmacy, 700115 Iasi, Romania; antigona.trofor@umfiasi.ro (A.C.T.); cristian.cojocaru@umfiasi.ro (C.C.); 3 Faculty of Agriculture, Food Technologies Department, Life of Sciences University “Ion Ionescu de la Brad”, 700490 Iasi, Romania; r_stela_222@yahoo.com

**Keywords:** asthma, remission, pathways

## Abstract

**Background**: Asthma is a chronic inflammatory disorder in millions of individuals across the globe with high morbidity, mortality, and health care costs. Despite advances in asthma treatment, long-term remission is a challenging target to achieve. **Objectives:** This review will address the path to remission in asthma with focus on the role of biologic agents in severe asthma management and on the question as to whether long-term disease control and remission are a reality. **Methods**: A systematic literature review from 1971 to 2025 was conducted through databases such as PubMed, MEDLINE, Scopus, and Web of Science. Clinical trials, meta-analyses, and real-world evidence concerning biologic therapies, such as monoclonal antibodies targeting interleukin -5 (IL-5), IL-4/IL-13, immunoglobulin E, and thymic stromal lymphopoietin, were considered. Symptom control, exacerbation frequency, lung function, and oral corticosteroid (OCS) use were some of the outcomes considered. **Results**: Biologic treatments have yielded significant gains in asthma control and reduction of exacerbation. Complete remission—long-term resolution of symptoms, inflammation, and drug dependence—is still difficult to achieve. Early intervention with biologics may prevent irreversible airway remodeling, but long-term remission is not in sight. These drugs reduce OCS dependency, but sustainability of remission remains to be investigated. **Conclusions**: Biologic therapies have advanced asthma treatment, particularly in severe cases, by improving symptoms and reducing exacerbations. However, complete remission remains a distant goal. The development of standardized remission criteria, better patient stratification, and long-term clinical studies are necessary to help achieve sustained asthma control and remission.

## 1. Introduction

Asthma is a long-term inflammatory airway disease that affected more than 339 million people worldwide in 2020 and accounted substantially for morbidity, mortality, and healthcare costs [1]. Asthma caused approximately 455,000 deaths globally in 2019, according to the World Health Organization, and it is a frequent reason for avoidable hospitalization, as well as a source of economic burden for healthcare systems [2]. There are still a large number of asthmatic patients with exacerbations, and impaired quality of life in spite of improvements in disease management, leading to high healthcare access and socioeconomic burden [3]. The Global Asthma Report 2022 estimates that asthma prevalence was 9.1% in children, 11.0% in adolescents, and 6.6% in adults, with regional disparities influenced by environmental, genetic, and healthcare access factors [4].

Traditional treatment of asthma has been focused on symptom control with bronchodilators and inhaled corticosteroids (ICS), which are very effective in suppressing airway inflammation and preventing exacerbations. Treatment does not change the disease process, however, and patients are still reliant on chronic pharmacotherapy and susceptible to relapse of symptoms [5]. This limitation has created the possibility of developing a framework for the definition of asthma remission, around disease stability, reducing exacerbations and reducing drug dependence [6].

Remission in asthma has, however, been a priority over the years in treatment. However, real remission, as long-term symptom-free, inflammation-free, and exacerbation-free status, is still a mirage. Severe asthma pathophysiology is essentially Type 2 (T2) inflammation-dominated, with the major cytokines, interleukin-4 (IL-4), IL-5, and IL-13 involved. This inflammation results in eosinophilic airway inflammation, mucus hypersecretion, and airway remodeling, making it challenging to achieve permanent remission [7,8]. Airway inflammation is further exacerbated by epithelial-derived cytokines, like IL-33 and thymic stromal lymphopoietin (TSLP), which increase both eosinophilic and non-eosinophilic pathways [9]. Remission is therefore hampered by airway remodeling, which is a result of these inflammatory cascades.

The recent developments in precision medicine, and the advent of biologic treatments, have dramatically enhanced asthma outcomes, particularly in severe steroid-resistant patients. Monoclonal antibodies against anti-Interleukin-5 Receptor, anti-IL-5R (mepolizumab, reslizumab, benralizumab), IL-4/IL-13 (dupilumab), and TSLP (tezepelumab) have proven their efficacy in suppressing exacerbations, enhancing lung function, and reducing oral corticosteroid (OCS) reliance [6]. However, while these therapies enhance disease control, they do not yet induce true remission, as discontinuation often results in symptom recurrence [10]. Long-term asthma remission can necessitate early treatment prior to the onset of irreversible airway remodelling, emphasizing the importance of predictive biomarkers and phenotypic stratification in clinical practice.

Along with pharmacologic management, non-pharmacologic interventions may promote remission of asthma by modifying predisposing risk factors and immune mechanisms. Pulmonary rehabilitation has been found to be beneficial in improving airway mechanics and reducing frequency of exacerbation, particularly in obese asthma [11]. Allergen avoidance strategies have demonstrated partial success in reducing asthma severity, though long-term benefits remain controversial [12]. In addition, weight loss interventions among obese asthma patients are associated with improved lung function and reduced airway inflammation, which suggests that metabolic pathways play a role in asthma persistence [13]. Additionally, early-life interventions, including maternal health optimization, microbiome-targeted therapies, and controlled environmental exposure, have been shown to influence immune system development and reduce asthma severity in the long term [14]. However, while such approaches show promise, further research is necessary to determine if they induce true remission or merely improve symptom control.

This review assesses the current state of remission in asthma, focusing on the role of biologic therapies toward durable control of asthma. It attempts to address some of the most crucial variables that can plausibly bring about remission, including initial treatment, prognostic biomarkers, and the impact of airway remodeling on therapeutic response. Although remission from asthma remains a challenging and complex goal, ongoing research promises greater customized treatments that will move the science closer to its ideal of long-lasting remission in asthma sufferers.

## 2. Materials and Methods

A systematic search for remission of asthma was conducted on PubMed/MEDLINE, Scopus, and Web of Science. Clinical trial, meta-analysis, and clinical study filters were used with the key-words “remission” and “asthma”. The dates of search were 1971–2025, and articles were restricted to English language publications. MeSH vocabulary and Boolean connectors (i.e., “asthma”[MeSH] AND “remission”[MeSH] or asthma AND remission) were applied where appropriate in order to ensure completeness.

Articles were selected based on the subject of remission in asthma, i.e., articles investigating monoclonal antibodies to IL-5, IL-4/IL-13, IgE, and TSLP. Clinical trials, meta-analyses, and real-world evidence were considered if they documented the efficacy of biologic therapy in the control of asthma. The inclusion criteria focused on studies in which outcomes such as symptom control (e.g., Asthma Control Test, ACT), exacerbation rates, lung function (e.g., Forced Expiratory Volume in 1 s, FEV1), and reduced OCS use were reported. Non-asthma illness, non-human participants, and non-traditional interventions, such as acupuncture or herbal medicine, were the exclusion criteria for studies. Studies not using standardized or clinically accepted definitions of remission from asthma were also excluded.

First search retrieved 182 papers that were filtered via title and abstract. Following the exclusion and inclusion criteria, 162 articles were excluded: 43 studies involved non-human subjects, 39 studies used non-conventional interventions, such as herbal medicine, acupuncture, or homeopathy, 31 studies assessed non-asthma conditions (e.g., chronic obstructive pulmonary disease, eosinophilic disorders), 28 articles measured biomarkers but not clinical remission outcomes, and 21 studies had inconsistent or poor remission definitions that were not consistent with established well-known clinical guidelines.

**Figure 1:**
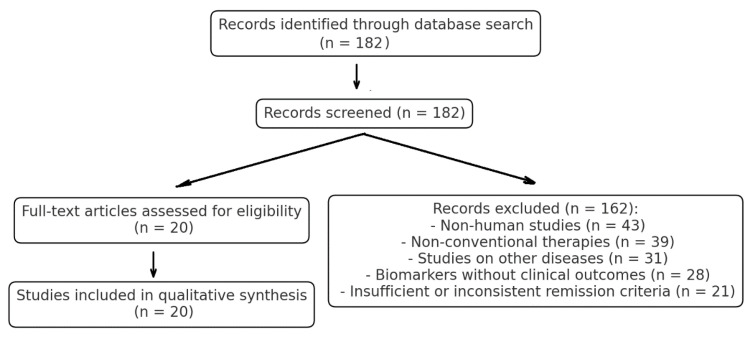
PRISMA flowchart detailing study selection.

Screening and selection were performed independently by two reviewers, and disagreement was resolved by discussion and consensus. The remaining articles were tested for full-text eligibility, and the final inclusion of 20 studies was made in the qualitative synthesis. Study selection followed the Preferred Reporting Items for Systematic Reviews and Meta-Analyses (PRISMA) guidelines, and the entire screening process is illustrated in the PRISMA flowchart (Figure 1).

## 3. Defining Asthma Remission: Conceptual Framework and Expert Consensus

Remission in asthma is a concept of interest, with newer therapies, particularly biologic agents, offering new hope for long-term disease control. Remission in asthma is defined in multifaceted and varying ways among studies and clinical practices. Traditionally, remission in asthma has been defined by the chronic absence of symptoms, but the use of this one criterion is insufficient, considering the chronic condition and irreversible airway structural changes occurring even in patients without symptoms. In this context, the concept of remission has become ever more prominent. Although far from a novel phenomenon, remission has been described in 52–75% of children with asthma, and adolescence is the most common period for spontaneous remission of symptoms [15]. In adults, the evidence available suggests that 2–25% of patients may undergo spontaneous remission of their disease, but the estimates vary according to population studied and criteria for remission [16].

Despite the growing interest, asthma remission remains a controversial concept. Even though remission does not imply cure, it generally defines the long-term absence of the disease symptoms over a prolonged period of time. One of the most significant areas of uncertainty is whether remission should involve normalization of lung function, because certain aspects of bronchial dysfunction are not reversible. Following airway remodeling, ventilatory impairment becomes permanent. An association between a declining peak expiratory flow (PEF) and yearly exacerbation rate has also been proposed [17]. Longitudinal studies have shown that, over a 20-year follow-up, PEF deterioration is worse in young patients and in patients with frequent exacerbations, and the importance of early intervention in both preventing structural airway change and reducing the psychological effect of asthma has been emphasized [17,18].

In order to achieve standardization of definitions and consensus, a Delphi survey was conducted [19], which led to the proposal of four distinct remission frameworks: clinical versus complete remission, each of which may be present with or without treatment. This classification provides a more structured method of assessing asthma remission in research and clinical practice. A definite notion of clinical versus complete remission is required [20].

Clinical remission would typically be defined by the recurring absence of symptoms, as defined by validated tools like the ACT, and normal lung function over a prolonged period of time (typically 12 months). Clinical remission would typically also include no requirement for OCS therapy. This is an improvement definition that encompasses symptom improvement and quality of life but not always complete reversal of airway injury or inflammation.

Complete remission, on the other hand, is more than the suppression of symptoms, to include normalization of airway function and inflammation. This is provided by the Spanish Consensus on Remission in Asthma (REMAS) consensus, which operationalizes remission based on the absence of both systemic and bronchial inflammation. REMAS-defined remission is characterized by Fractional Exhaled Nitric Oxide, FeNO <40 ppb, sputum eosinophils <2%, negative bronchial hyperresponsiveness, and no evidence of bronchial remodeling [15]. This formalizes and gives a more comprehensive definition of asthma remission, enabling improved research and clinical practice.

Complete remission would imply not only the absence of symptoms but also the absence of inflammation and structural changes in airways. However, so far, treatments developed have not managed to do so consistently, and complete remission is an area for future studies [21,22].

Follow-up consensus panels have subsequently further defined these principles, suggesting that asthma remission should be quantified on the basis of a composite of clinical events and objective evidence of inflammation and lung function. Such standards would better delineate complete and clinical remission and could both guide clinical practice and target asthma care research in the future (Table 1).

**Figure 2:**
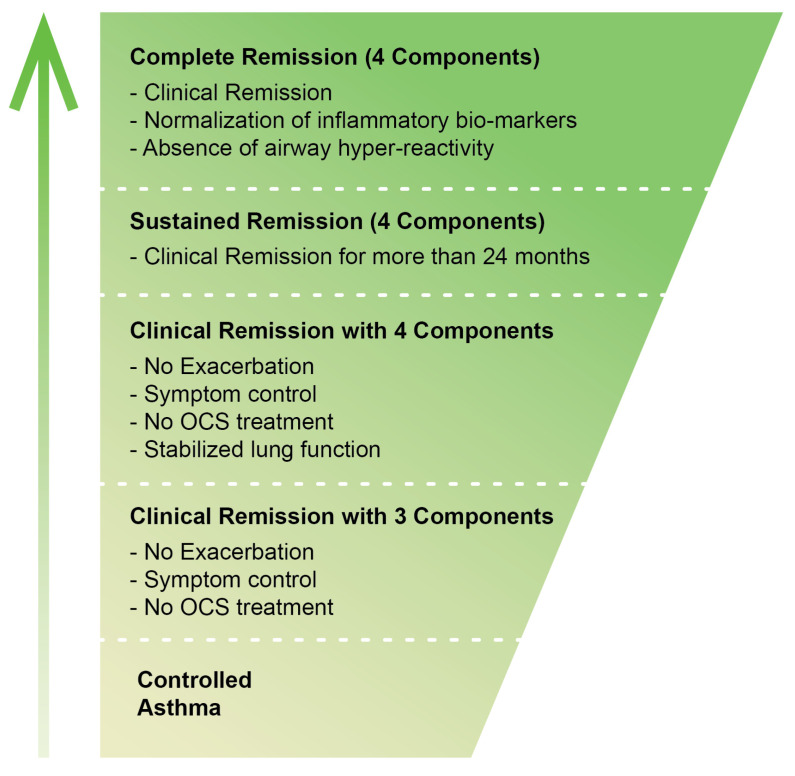
Stepwise Progression of Asthma Remission; OCS, oral corticosteroids. The green gradient illustrates the progressive improvement in asthma status, ranging from controlled asthma at the base (light green) to complete remission at the top (dark green).

Another emerging concept of interest to researchers and clinicians is sustained remission. Currently, this state is defined by the persistence of clinical remission criteria for up to 24 months of follow-up (Figure 2).

## 4. Pathophysiology of Asthma and Therapeutic Targets for Remission

Asthma is a complex disease with intricate etiology and pathophysiology which govern the pattern of the disease, as well as its potential to go into remission. Chronic airway inflammation has even been observed in remission, which may lead to the recurrence of symptoms, airway hyperresponsiveness (AHR), and bronchial remodeling. The mechanisms of chronic inflammation, airway hyperresponsiveness, and structural remodeling must be understood in order to develop therapeutic targets which can induce remission in chronic asthma [23].

### 4.1. Chronic Inflammation and Immune Dysregulation

The asthmatic inflammatory cascade begins with the antigen-presenting cells (APCs), which, together with other immune cells, such as eosinophils and neutrophils, secrete signature cytokines, such as IL-4, IL-5, and IL-13. Elevated levels of IL-4 are responsible for the persistence of the immune response, particularly by facilitating the interaction between the T and B cells, such that IgE synthesis is enabled, and the activation of mast cells. Basophils also add to this by stimulating cytokine production, perpetuating the inflammatory response. This prolonged exposure of APCs to T cells maintains the chronic inflammatory process leading to bronchial muscle hypertrophy, mucus hypersecretion, and irreversible airway obstruction, all the characteristic features of airway remodeling. Even when in remission and inflammation might resolve, studies have indicated that inflammatory markers remain elevated compared to healthy controls. Histopathological analysis found that basement membrane thickening, a characteristic of severe asthma, is not fully reversed during remission. This implies that, while the inflammatory burden can be alleviated, structural alterations remain and patients may remain at risk of disease recrudescence [24].

### 4.2. AHR and Its Role in Remission

AHR is one of the features of asthma and continues to be of clinical significance even in remission. AHR is not necessarily a part of formal remission criteria, though, mainly because of the restricted accessibility of bronchial challenge testing, is usually available in specialized centers. In spite of this, research has established that around 57% of clinically remitted patients continue to present with bronchial hyperresponsiveness [25]. This would, in turn, necessitate more defined criteria for remission, as persistent AHR might indicate continuing inflammation and heightened risk of exacerbations and progression of the disease. Thus, AHR, even when asymptomatic clinically, may well become an important predictor of subsequent exacerbations and an obstacle to attaining the real remission state.

### 4.3. Airway Remodeling and the Impact on Remission Potential

Airway remodeling is a major obstacle to the remission of asthma. Chronic inflammation, together with genetic predisposition and frequent exacerbations, results in structural airway changes. Such changes, which involve fibrosis, smooth muscle hypertrophy, and goblet cell hyperplasia, correlate with asthma severity and duration and are a key determinant of the likelihood of remission. The extent of such structural changes predicts the possibility of remission, since these changes are not reversible and persist even when there is no active inflammation.

Evidence exists that the chance of spontaneous remission reduces by around 15% for every 10 years of asthma onset. This is in favor of early control of the disease in order to avert permanent airway damage [26]. In individuals with long-standing remodeling of the airways, complete remission is less likely, and more intense therapy is needed to reverse inflammation, maintain lung function, and even stop or reverse airway structural changes.

## 5. Current Strategies to Achieve Remission

Clinical or complete remission of asthma is still a puzzling dilemma. It will require the combined application of both pharmacologic treatment regimens and non-pharmacological interventions, such as psychological therapy, weight management, and removal or alteration of environment and lifestyle-related precipitants. Total remission of asthma continues to elude the majority of patients despite all these measures, since remodeling within the airway and persistent inflammation remain challenges to disease remission in the long term.

**Figure 3:**
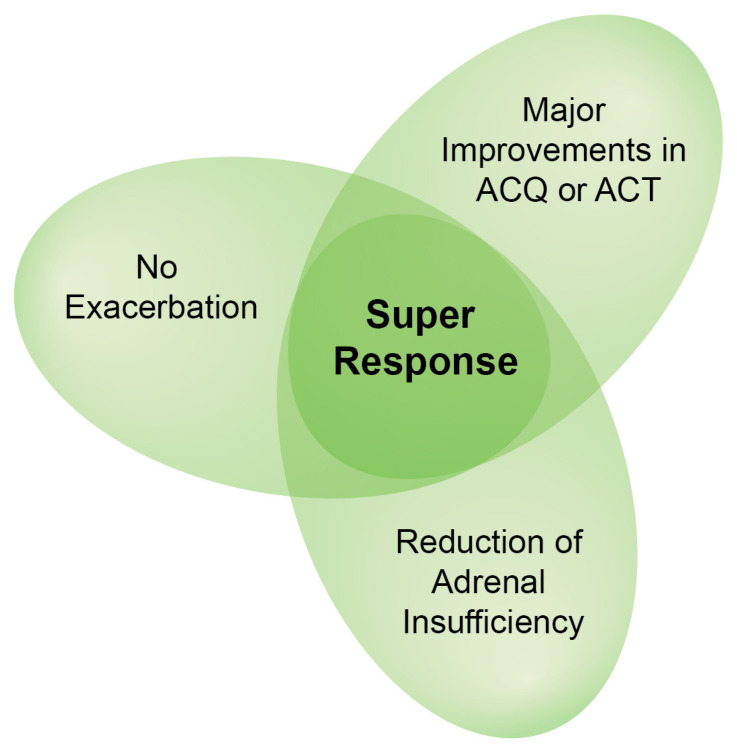
Criteria for Super Response in Asthma Treatment. Shades of green indicate the overlap of clinical improvements, with the darkest area representing a “Super Response” where all three criteria are met.

The term “super-responders” has been used, referring to a subset of patients with a rapid, persistent, and favorable response to initiation of biologic therapy. Such patients meet certain remission criteria within 12 months, but identification of this subset is still challenging (Figure 3). Additional research needs to be carried out to establish predictors for such favorable responses and determine more specific asthma remission criteria.

### 5.1. Pharmacological Interventions

Pharmacotherapies, in the form of monoclonal antibodies directed against central cytokines involved in asthma pathophysiology, have been successful in optimizing asthma control and lung function. True remission, by the irreversible disappearance of symptoms, inflammation, and airway remodeling, remains a distant goal. While therapeutic efficacy of biologic therapies has been promising, none of the above treatments have resulted in true asthma remission, although they have optimized symptoms control and reduced exacerbations (Table 2).

Omalizumab (Anti-IgE) was assessed in the PROSPERO real-life study, which demonstrated that 23% of patients treated with omalizumab for 12 months achieved significant improvements in ACT score (≥20) and FEV1 (increase >120 mL), with no exacerbations or need for OCS. However, while omalizumab improved asthma control, complete remission was not achieved, as symptoms often returned after the discontinuation of therapy [27].

Dupilumab Anti-interleukin-4 receptor α (Anti-IL-4Rα), investigated in the Liberty Asthma QUEST 2021 Phase 3 trial [28] and TRAVERSE open-label extension [29], produced similar results, where 31.7% of the subjects reached the endpoint of improved symptom control and better pulmonary function following 12 months of treatment. FEV1 was greatly improved, and Asthma Control Questionnaire (ACQ-5) scores were below 1.5, demonstrating effective control of asthma symptoms without exacerbations.

Benralizumab (Anti-IL-5Rα), attempted in several trials, including the Phase 3 SIROCCO/CALIMA trials [30] and the Phase 3b ANDHI trial [31], yielded less uniform results. In the SIROCCO/CALIMA trials, 23.9% of the patients achieved the study’s main endpoint at 12 months, with both improvement in FEV1 and symptoms. On the other hand, the ANDHI trial reported a lower success rate of 16.6% at 6 months with great FEV1 improvements but quite modest relief in symptoms. The XALOC-1 real-life study [32] had a duration of 12 months, with 43% of patients achieving the treatment aim.

Reslizumab (Anti-IL-5), investigated in 2022 real-life research, showed 40% patients having symptom control and pulmonary function up to 12 months. It was proven that reslizumab could induce dramatic control of asthma, with ACT score improvement and FEV1 (2022 Real life) [33].

Mepolizumab (Anti-IL-5), via the REDES real-life study analysis, showed that 30% of patients reached the goal of symptom control and ideal pulmonary function at 12 months. The study was shown to have notable exacerbation reductions and FEV1 improvement, with the importance of mepolizumab in treating severe asthma being determined (REDES) [34].

Tezepelumab (Anti-TSLP), which was evaluated in the NAVIGATOR Phase 3 trial, had the highest success rate, where 46% of the patients had attained the treatment outcomes at 12 months. This included significant improvement in FEV1 and asthma control, proving the therapeutic effectiveness of tezepelumab in severe asthma [35].

Overall, while all of the monoclonal antibodies were successful in improving asthma control and lung function, rates of patients who met target responses varied. The most efficacious were tezepelumab, followed by dupilumab and reslizumab. None of the treatments resulted in actual asthma remission, and more studies are required to optimize methods of treating asthma and to establish best therapies for diverse groups of patients.

**Table 2:** Study characteristics.

Monoclonal Antibody	Study Name	Absence of Symptoms	Optimized/Stabilized Pulmonary Function	No Exacerbations/No OCS	Duration	Aim Achieved by
Omalizumab Anti-Ig E	PROSPERO Real life [27]	ACT ≥ 20	FEV1 increase ≥ 120 mL	followed-up	12 months	23%
Dupilumab Anti-IL-4Rα	Liberty Asthma Quest 2021 Phase 3 [36]	ACQ-5 < 1.5	FEV1 post-BD ≥ 80%	followed-up	12 months	31.7%
	TRAVERSE open-label extension [29]	ACQ-5 < 1.5	FEV1 post-BD ≥ 80% or FEV1 pre-BD increase ≥ 100 mL	followed-up	12 months	31.7%
Benralizumab Anti-IL5Rα	SIROCCO/ CALIMA Phase 3 [30]	ACQ-5 < 1.5 or ≤ 0.75	FEV1 pre-BD increase ≥ 100 mL	followed-up	12 months	23.9%
	ANDHI Phase 3b [31]	ACQ-5 < 1.5 or ≤ 0.75	FEV1 pre-BD increase ≥ 100 mL	followed-up	6 months	16.6%
	XALOC-1 Real life [32]	ACQ-5 < 1.5 or ≤ 0.75	No data	followed-up	12 months	43%
Reslizumab Anti-IL5	2022 Real life [33]	ACT ≥ 20	No data	followed-up	12 months	40%
Mepolizumab Anti-IL5	REDES Real life [34]	ACT ≥ 20	FEV1 post-BD ≥ 80%	followed-up	12 months	30%
Tezepelumab Anti-TSLP	NAVIGATOR Phase 3 [35]	ACQ-5 ≤ 0.75	FEV1 pre-BD > 80% or increase FEV1 pre-BD > 20%	followed-up	12 months	46%

Legend: OCS—oral corticosteroids; Ig E—immunoglobulin E; ACT—Asthma Control Test; FEV1—forced expiratory volume in one second; IL-4Rα—interleukin-4 receptor α; ACQ—Asthma Control Questionnaire; BD—bronchodilator; IL-5Rα—interleukin-5 receptor α; IL-5—interleukin-5; TSLP—thymic stromal lymphopoietin.

The development of biologic therapies has opened new therapeutic opportunities in asthma. Identifying the most appropriate pathogenic pathways—by inhibiting IL-5 in patients with eosinophilic phenotypes, IL-4/IL-13 in severe T helper 2 (Th2)-type asthma, or IgE inhibitors for allergic asthma—has led to substantial progress in disease control, with broader possibilities for blocking the inflammatory process.

#### 5.1.1. IL-5 Inhibitors

IL-5 inhibitors have now emerged as effective therapies in eosinophilic asthma through inhibition of IL-5 binding to its receptor on eosinophils, leading to the reduction of blood eosinophil levels. The development of three principal biologics—mepolizumab and reslizumab (anti-IL-5 monoclonal antibodies) and benralizumab (anti-IL-5R) monoclonal antibody)—has been promising in the management of eosinophilic asthma. These therapies are well-tolerated and have been shown to reduce exacerbations and reduce the use of systemic corticosteroids (SCS) in patients with refractory eosinophilic asthma.

Eosinophils induce airway damage via cytokine secretion, macrophage, mast cell, and dendritic cell activation, inducing a persistent cycle of inflammation and inciting bronchial hyperresponsiveness, mucus secretion, and airway remodeling [37].

Activation of eosinophils in the respiratory system is central to the pathophysiology of asthma and, through prevention of recruitment and activation of eosinophils, IL-5 inhibitors interrupt the cascade of inflammation. The treatments also act by decreasing eosinophilic infiltration, as well as triggering antibody-dependent cellular cytotoxicity, leading to a reduction of eosinophils.

A Phase I multicenter benralizumab trial had a 95.8% reduction of eosinophils in the bronchial mucosa following 12 weeks of treatment at 100 or 200 mg versus baseline [38]. Consequently, conditions are created to achieve clinical or even complete remission, as evidenced by the absence of exacerbations, the elimination of OCS, and improvements in ACQ and ACT scores.

A post hoc analysis of the SIROCCO/CALIMA studies, in which patients were corticosteroid-naive at baseline, as well as the ZONDA study, which included patients who had received less than 12.5 mg of OCS, aimed to achieve remission as assessed by an ACQ-6 score <1.5 or ≤0.75, a pre-bronchodilator FEV1 increase of over 100 mL, and the absence of exacerbations and OCS use (Table 2) [30]. Treatment with benralizumab led to clinical remission, as measured by four criteria, in 23.9% of patients in the SIROCCO/CALIMA studies (ACQ score < 1.5). When two remission components were considered in the analysis, 87% of patients met the criteria for analysis. Given that the inclusion criterion for these studies was severe asthma requiring high doses of ICS/LABA, the results are promising [30].

Analysis of data from the ANDHI trial, which used the same remission criteria over a 6-month period, revealed that 16.6% of participants met all remission criteria, despite being high exacerbators at the time of study enrollment while on high-dose ICS therapy [31].

The XALOC-1 study analyzed real-world, retrospective data from 1002 patients with severe eosinophilic asthma who received benralizumab treatment. The follow-up criteria included results from the ACQ or ACT questionnaires, as well as the absence of exacerbations and OCS use [32]. Over one-third of the patients included in this study (37.9%) had received other biologic therapies during the baseline period, suggesting that they belonged to the category of lack responders.

Another real-life study, which examined the effects of reslizumab (an anti-IL-5 monoclonal antibody), demonstrated that, at the end of the 12-month follow-up period, 40% of patients with severe eosinophilic asthma achieved the study’s objectives: absence of exacerbations, no use of OCS, and an ACT score ≥20 [33]. Pulmonary function was not monitored, and clinical remission was assessed based on three criteria. Similar patients were included in the REDES real-life study, where mepolizumab was administered. At the end of the 12-month study period, 30% of patients met four remission criteria, and 37% met three criteria [34].

#### 5.1.2. Dual Blockade of IL-4 and IL-13

IL-4 and IL-13 are key asthma pathogenetic cytokines and are both implicated directly in bronchial inflammation and airway remodeling. IL-4 and IL-13 cause epithelial barrier dysfunction, goblet cell hyperplasia, smooth muscle and mucus cell proliferation, fibrosis, and eosinophilic infiltration of the lung. IL-4 and IL-13 also increase eosinophil chemo-attractants, such as vascular cell adhesion molecule (VCAM) and eotaxin, which are crucial for eosinophils migrating out of the blood and into airway tissue [39,40]. Additionally, through B cell activation, IL-4 and IL-13 cause release of inflammatory mediators, such as leukotrienes, histamine, and prostaglandin D2 (PGD2), from mast cells and basophils and thereby enhance inflammation [41]. By binding to the IL-4Rα subunit, dupilumab inhibits IL-4 and IL-13, thereby reducing inflammatory mediators, such as FeNO, eotaxin-3, total and specific IgE, thymus and activation-regulated chemokine (TARC), and periostin [42]. Inhibition leads to reduced airway inflammation, lung function improvement, and reduction in rates of severe asthma exacerbations, proving that it can induce remission in asthma patients.

QUEST study findings revealed that 31.7% of the moderate-to-severe uncontrolled asthmatic patients treated with dupilumab for 52 weeks were in clinical remission, versus 17.7% of patients in the placebo group [43]. In additional analysis by the TRAVERSE study of 1227 patients from the QUEST cohort, 36.4% of patients were in clinical remission at 100 weeks of treatment. Interestingly, post hoc analysis showed that 70% of patients were in long-term clinical remission and the rate of non-improvers diminished with time. This suggests that dupilumab is capable of producing long-term sustained benefit in the management of asthma and further suggests its use in long-term asthma control [43].

#### 5.1.3. Anti-IgE Therapy

Omalizumab is a humanized IgE monoclonal antibody that plays a critical role in the management of allergic asthma, since it acts against IgE, the key mediator of the allergic inflammatory process. It acts by binding to the IgE molecules, the Cε3 domain, and thereby inhibiting them from binding to the high-affinity IgE receptor (FcεRI) on mast cells, basophils, dendritic cells, and eosinophils. This blockade decreases mast cell and basophil activation and degranulation, and consequent release of pro-inflammatory mediators, such as histamine and leukotrienes. Additionally, by blocking IgE-mediated activation, omalizumab modulates the immune system and decreases overall eosinophilic airway activity, characteristic of type 2 (T2) asthma inflammation.

Clinically, omalizumab has been demonstrated to decrease asthma symptoms and frequency of exacerbations, and to enhance lung function. This is especially so for severe allergic asthma patients, in whom control cannot be achieved with standard inhaled therapy. Omalizumab therapy has repeatedly been demonstrated in trials to decrease the requirement for OCS, with a steroid-sparing effect, which is extremely useful in avoiding the side effects of corticosteroids in the long term [44,45,46].

A real-life PROSPERO study in patients with allergic asthma showed that 23% of patients who were given omalizumab for 48 weeks had clinically relevant improvements, including an ACT of ≥20, an increase in FEV1 of >120 mL, and no exacerbations. These findings show that omalizumab significantly improves asthma control and lung function and reduces the rate of exacerbations and the consumption of OCS. Despite these positive outcomes, omalizumab does not induce actual remission, because symptoms will relapse upon withdrawal of treatment [27].

These findings have been substantiated by various studies and systematic reviews, and omalizumab has proved to be a useful treatment for severe allergic asthma. However, as in other biologic therapy, full remission of asthma, i.e., long-term freedom from symptoms, inflammation, and airway remodeling, with omalizumab has not been documented. Therefore, even though omalizumab results in remarkable symptom control and lung function improvement, it remains part of an extended therapeutic approach for asthma management rather than for remission.

#### 5.1.4. Anti-TSLP

The potential of anti-TSLP therapy to induce remission in asthma is a new and promising field of research, particularly in resistant and severe asthma. Thymic stromal lymphopoietin (TSLP) is an epithelium-derived cytokine with a key role in the initiation and amplification of asthma inflammation. Its inhibition represents a new therapeutic strategy for the treatment of asthma, particularly in those patients who are non-responders to conventional therapies. Tezepelumab, a monoclonal antibody against TSLP and TSLP-blocking, was effective for asthma exacerbations and improved asthma control, with a suggestion of a pathway towards remission in a subgroup of patients [47].

TSLP is released as a result of epithelial injury and inflammation of the bronchial epithelium and thereby becomes central to the pathogenesis of Type 2 (T2) inflammation in asthma. It triggers a cascade of immune events, orchestrating dendritic cell, T and B cell, eosinophil, basophil, mast cell, neutrophil, and macrophage activation, all of which participate in airway inflammation, remodeling, and bronchial hyperresponsiveness. Through this mechanism, TSLP directly regulates airway remodeling alterations, such as goblet cell hyperplasia, smooth muscle hypertrophy, and fibrosis, that are features of asthma and are major barriers to the achievement of remission. Tezepelumab inhibits TSLP through binding to it, its interaction with the receptor, and thus the release of inflammatory mediators. Through TSLP inhibition, eosinophil activation and airway inflammatory burden are decreased. In Phase 2 and 3 trials, tezepelumab has been found to be safe and effective in severe asthmatics with clinically significant exacerbation and hospitalization rates and need for SCS [47].

In the Phase 3 NAVIGATOR trial, 46% of patients with severe uncontrolled asthma who received tezepelumab reached clinical remission status at the end of follow-up, which reflects its capacity to induce sustained improvement in asthma control and potentially lead to remission [48]. Significantly, tezepelumab was effective in a broad set of asthma phenotypes, including both eosinophilic and non-eosinophilic asthma, showing its versatility and potential in managing heterogeneous asthma patient populations.

Furthermore, TSLP concentrations in sputum have also been identified as a promising predictive biomarker of remission in patients with severe eosinophilic asthma. The finding that TSLP concentrations have been described as a predictive biomarker also suggests that measurements of TSLP levels would not only determine which patients will respond to treatment with tezepelumab or anti-IL-5 but, as such, also allow personalized treatment. The commonality of the action of anti-TSLP and anti-IL-5 treatment in preventing eosinophilic inflammation also provides the rationale for combination therapy in enhancing severe asthma outcomes [49].

Further, novel humanized anti-TSLP monoclonal antibodies, such as TAVO101, are also being developed for use in the treatment of TSLP-related disorders, such as asthma and psoriasis. TAVO101 was a highly effective TSLP neutralizer and showed promising pharmacokinetic profiles in preclinical models, suggesting that it represents a promising candidate in the therapeutic arsenal for the treatment of asthma and other inflammatory diseases [50].

#### 5.1.5. Emerging Treatments

##### JAK Inhibitors

Janus kinase (JAK) inhibitors are being evaluated in the treatment of various inflammatory diseases, including asthma, for their ability to modulate numerous cytokine pathways involved in disease pathophysiology. The JAK family comprises JAK1, JAK2, JAK3, and Tyrosine Kinase 2 (TYK2), all of which play significant roles in the signal transduction of numerous pro-inflammatory cytokines, like IL-4, IL-5, IL-6, IL-9, IL-13, granulocyte-macrophage colony-stimulating factor (GM-CSF), interferons (IFN-α, IFN-β, IFN-γ), and TSLP. These cytokines participate in the inflammatory processes of asthma, namely in Type 2 (T2) inflammation, which is associated with eosinophilic and allergic asthma [51].

A study confirmed that tofacitinib, a JAK inhibitor, suppressed IL-5 production in monotherapy and combined therapy with dexamethasone. Further, tofacitinib suppressed phosphorylation of chemokine ligand 5 (CCL5), with the effect increased through the combination of the drug with dexamethasone. The findings suggest that JAK inhibitors will prove particularly effective in corticosteroid-resistant asthma, where conventional drugs are ineffective in suppressing symptoms [52]. By suppressing chemokine production by epithelia, JAK inhibitors may be a new choice for asthma treatment, with reduced inflammation and shielded additional damage in airways.

JAK inhibitors tofacitinib and baricitinib have also come under examination for the prevention of bronchial remodeling through restoration of epithelium’s barrier function, which is central to airway protection and immunity. JAK2 antagonists would be ideal for eosinophilic asthma, since they would inhibit the activation of eosinophils, which are the central propagators of pulmonary inflammation. These therapies have a potential benefit over IL-5 inhibitors in directly targeting eosinophils, in the sense that they produce more modulation of the inflammatory cascade with inhibition of IL-4 and IL-13 signaling.

##### Prostaglandin D2 Receptor Antagonists

Prostaglandin D2 (PGD2) and the chemoattractant receptor-homologous molecule expressed on TH2 cells (CRTh2), its receptor, have both been implicated in the pathogenesis of allergic and eosinophilic asthma. CRTh2 activation produces high intracellular concentrations of calcium as well as low cyclic adenosine monophosphate (cAMP) concentrations. Both of these are crucial for the activation of eosinophils and basophils. Inhibition of CRTh2 activation has been proposed as a treatment strategy for asthma inflammation by inhibiting the release of critical cytokines, including IL-5, IL-4, IL-13, IL-33, and IL-25, and blocking eosinophil and basophil degranulation. It may restore the integrity of the bronchial epithelium and prevent airway injury due to chronic inflammation [53].

Experimental studies, in vivo and in vitro, have shown that CRTh2 antagonism can reduce bronchial hyperreactivity and inflammation remarkably. CRTh2 inhibition has led to reduced eosinophilic and basophilic lung infiltration, reduced IgE in the blood, and reduction of pro-inflammatory cytokines in bronchoalveolar lavage. These findings are compatible with the hypothesis that CRTh2 receptor antagonists can treat salient features of asthma pathogenesis, i.e., in eosinophilic or Th2-dominant asthmatic patients [54].

A study has determined the role of PGD2 metabolites in causing secretion and migration of IL-5 and IL-13 and morphological remodeling of eosinophils via CRTh2 activation. The PGD2 metabolites also promoted Type 2 innate lymphoid cell (ILC2) migration, which plays a crucial role in airway inflammation. These observations indicate that CRTh2 antagonists not only suppress eosinophil and basophil activity but also suppress the recruitment of ILC2 cells, which are believed to contribute to the chronicity of asthma [55].

Genetic studies have shown that specific genetic polymorphisms are associated with potential causative overexpression of CRTh2 and hence an increased inflammatory response towards asthma. The findings are indicative of the therapeutic potential of CRTh2 blockade as a highly targeted strategy for individualized treatment of asthma, specifically in individuals with overexpression of CRTh2. The same population of patients could be characterized by genetic profiling so that treatment could be stratified and therapy maximized with CRTh2 antagonist medications.

## 6. Research and Future Directions

Progress in the molecular and genetic underpinnings of asthma, and the phenotyping of various forms, has encouraged the development of new therapies that have significantly improved quality of life and outcome for the patient. However, while this progress has been achieved, there are certain avenues of research that remain most important in the effort to treat asthma. Research in these areas is potentially capable of revealing new therapeutic strategies, to bring additional benefit to the patient and to ultimately lead to long-term remission.

### 6.1. Microbiome Research

The microbiota plays a direct role in managing immune system homeostasis and providing protection to the respiratory tract from infection. Dysbiosis has been shown to induce inflammation in the airway by seeding the airway with microbes that are capable of inducing a pro-inflammatory state. Particularly, *Proteobacteria phylum* has been identified as being pivotal in inducing this dysbiosis, resulting in exacerbation of asthma symptoms [56]. There has been mounting evidence of the interaction between the respiratory and gastrointestinal microbiota and the circulating metabolites in the blood linking the two microbiomes. Probiotics have been implicated by some studies to be central to the relief of the severity of asthma through rebalancing of the microbiota [57]. It has been observed that infants with higher butyrate and propionate levels in feces—usually resulting from supplementation of fiber—have lower atopy and eosinophils in sputum, reflecting a beneficial effect on asthma management. Additionally, the severity of asthma has been correlated with the presence of histamine-producing bacteria, once again reflecting the role of the microbiome in asthma pathogenesis.

### 6.2. Acute Asthma Event Prediction

It is now possible, due to recent advancements in predictive modeling, to determine the risk factors of acute asthma exacerbations. Logistic regression-derived statistical models have been developed to forecast these events with respect to patient-specific variables. For example, age and being underweight were determined to be significant risk factors for exacerbation, and therefore individualized assessment of risk becomes a possibility when managing asthma [58]. These forecasting models hold the promise of enhancing decision-making algorithms by incorporating a variety of variables, such as physical traits, functional markers, immunological status, genetic factors, and even imaging or microbiological data. With the advent of sophisticated algorithms, predictability and accuracy of the predictions can be significantly improved. These algorithms would not only predict acute asthma attacks but also guide individualized treatment streams, including the most appropriate therapies for inducing remission.

In the future, more precise identification of at-risk patients will be expected as predictive models become more precise. With the possibility of artificial intelligence and machine learning, it will probably be possible to track asthma in real-time and dynamically tailor treatment based on real-time feedback. These advances will, in turn, potentially enable individualized treatment regimens, optimize patient benefit, and bring long-term remission of asthma closer.

As future directions, there is a requirement for inclusion of real-world clinical practice experience into asthma guidelines. This requires that recommendations be adjusted to encompass earlier treatment initiation with biologics among high-risk patients and a clearer strategy for long-term remission and biologic withdrawal. Real-world data can be used to improve more individualized and proactive treatment plans with the capacity to deliver enhanced long-term control of asthma.

## 7. Challenges in Achieving Remission

Asthma remission remains a multi-factorial objective, challenging to attain. Remission has been demonstrated as more likely to result when airway morpho-pathological changes are at an initial stage and have not yet progressed to airway remodeling. Intervention with novel therapeutic strategies, if timely, can potentially reduce patient exposure to therapies with significant multi-organ effects, such as SCS. From this perspective, remission appears more possible in mild or moderate asthma patients but is also possible in severe asthma patients, provided a personalized and integrated treatment approach is taken (Table 3).

However, there remain some hurdles in the search for true asthma remission. One of the key hindrances is the need for universal remission criteria. Current remission definitions are often heterogeneous, with variation in the perception of clinical remission between patients and clinicians. While patients might experience symptom control and reduced exacerbations, the achievement of complete remission—i.e., the lack of symptoms, inflammation, and airway remodeling—is a distant dream. Thus, a standardized remission definition framework is necessary to guide treatment and measure success in a broad spectrum of patient populations.

Most clinical trials that have tested new biologic therapies have enrolled patients with severe asthma, who are generally on high-dose ICS/LABA or OCS at present. As the patients enrolled are already at an advanced stage of the disease, achievement of remission in fewer than 50% of patients should not be the ultimate criterion for success. While these therapies may improve disease control and quality of life, the results suggest that the most severe asthma patients are unlikely to experience complete remission. This limited success highlights the need for a more tactical approach to asthma remission—one that focuses on early intervention to prevent disease progression and airway remodeling prior to their becoming established.

## 8. Discussion

The definition of “remission” in asthma has been widely discussed in the literature, and there is no consensus regarding the extent of improvement necessary to qualify a patient as being in remission. We recognize that the term “remission” is complex and context specific, since it may vary with the stage of the disease, type of intervention, and type of patient. In order to address this, we have referred to the most universally used definitions and classifications of asthma remission and highlighted the reasons behind its accomplishment.

As pointed out in the manuscript, we emphasized the fact that, while biologic therapies have made tremendous improvements in reducing symptoms and exacerbations, and improving lung function, their capacity to reverse bronchial remodeling is minimal when they are started at advanced stages of the disease. This is why we believe that starting biologic therapies earlier, particularly during the early stages of the disease when bronchial remodeling remains reversible, could lead to better outcomes in remission attainment.

Based on current knowledge, we are unable to estimate the remission duration in terms of time and symptoms after termination of therapy in routine practice. The current data reveal that there are 12–36 months of sustained remission [59]. Future studies will likely clarify the role of exogenous as well as endogenous variables in the potential loss of remission.

Complete remission is more than symptom resolution. This is indicative of a lack of inflammation, hyperreactivity of the bronchi, and normalization of the biomarkers, such as eosinophils and levels of FeNO. These are indicative of pathogenic mechanisms, in which eosinophils react to IL-5 activity and FeNO is an indicator of levels of IL-4 and IL-13 activity. Such biomarkers would be crucial to track in developing preventive measures. Treatment would then have to be tailor-made to treat those inflammatory pathways most predominant in a particular patient. This specific treatment may improve exacerbation management, pulmonary function, and disease course, such that complete remission is more probable.

However, clinical remission is extremely close to the definition of controlled asthma. It is acceptable to define clinical remission as having a minimum 12-month duration, considering the seasonality of asthma. The distinction is extremely important in the interpretation of the therapeutic goals because clinical remission may be the manifestation of a stable and well-controlled but not necessarily completely cured underlying inflammation. Therefore, while clinical remission can be seen as a tremendous success, complete remission should still remain the ultimate goal, signifying both symptom control and resolution of disease.

To the best of our knowledge, there are no predetermined criteria for stopping biologic therapy. Nevertheless, it is recommended that biologic therapy may possibly be stopped after 3–12 months’ duration after disease control [60]. One study highlighted that reasons given by patients and physicians for why treatment was discontinued were different, the most common reasons being lack of control and exacerbations [61]. The XPORT study showed that 47.7% of patients who had discontinued treatment with omalizumab were still well controlled during the first year of follow-up [62]. Another piece of research establishing the effect of withdrawing various biologics (omalizumab, dupilumab, mepolizumab, benralizumab, reslizumab) showed that 10.2% of the patients had a 50% or greater increase in severe exacerbations [60].

There is a close association between asthma and upper airway involvement, both characterized by a type 2 immune signature, which often results in severe and recurrent symptoms. As a result, patients with asthma and nasal polyposis are more challenging to control, experiencing more frequent exacerbations, bronchoconstriction, and heightened inflammation. In a clinical trial to assess the effect of tezepelumab in patients with chronic rhinosinusitis with nasal polyposis and uncontrolled asthma, there were remarkable changes in total nasal-polyp score and mean nasal-congestion score [63]. Similar results were reported in real-life retrospective observations in patients with severe asthma and chronic rhinosinusitis with or without nasal polyposis, treated with omalizumab, mepolizumab, benralizumab, or dupilumab. Improvement was seen after only 3 months of therapy in at least 80% of the patients, and all dupilumab-treated patients reached the minimal clinically important difference [64].

Atopic dermatitis is frequently associated with respiratory conditions, such as rhinitis, nasal polyps, and asthma. The interrelationships among these conditions are numerous and strong and relate to genetic predisposition, the Th2 phenotype, local immunity, and barrier dysfunction, among others. It has been estimated that the prevalence of asthma and atopic dermatitis will continue to rise until 2050, emphasizing the need to manage modifiable risk factors [65].

A holistic approach to managing severe asthma, including biologic therapy, multidisciplinary team management, personalized medicine, and patient-focused interventions, is needed to induce clinical remission. While there has been much progress, there remains much to be overcome, and further studies are needed to optimize outcomes in patients with severe asthma.

In the future, focus needs to shift away from managing end-stage, severe asthma towards the initiation of immunological therapy at an earlier stage in the disease process, with the possibility of preventing or reversing early structural changes in airways. This strategy would enhance the likelihood of achieving clinical and complete remission.

## 9. Limitations

The current research has several limitations, including the lack of evidence regarding the effectiveness of biologic treatments in early-stage asthma, where bronchial changes may still be reversible. There is no ideal agreement on remission criteria, especially between patients and physicians. Additionally, long-term follow-up data on remission after the discontinuation of biologic treatment are missing.

## 10. Conclusions

Asthma remains a major global health concern, with true remission still out of reach, particularly in severe cases. Biologic therapies, like omalizumab, dupilumab, benralizumab, tezepelumab, and reslizumab, significantly improve symptom control and lung function, but none have achieved complete remission, due to persistent airway remodeling and inflammation. Future research should focus on personalized treatment strategies, early intervention with immunological therapies, and the identification of predictive biomarkers, such as FeNO and eosinophil counts. By refining remission criteria and exploring combination therapies, asthma remission could become a more attainable goal, improving patient outcomes and reducing healthcare burdens.

## Figures and Tables

**Table 1:** Definitions of Asthma Remission in the Absence and Presence of Treatment.

Remission Type	No Treatment	During Treatment
Clinical Remission	Absence of symptoms	Absence of symptoms
	Optimization and stabilization of lung function	Optimization and stabilization of lung function
	Disease remission perceived by both patient and physician No use of OCS Maintained for more than 12 months	Disease remission perceived by both patient and physician No use of OCS Maintained for more than 12 months
Complete Remission	Criteria of clinical remission	Criteria of clinical remission
	Resolution of inflammation (reduction in eosinophil count, decreased FeNO levels) Absence of bronchial hyperreactivity	Resolution of inflammation (reduction in eosinophil count, decreased FeNO levels) Absence of bronchial hyperreactivity
Sustained Remission	Criteria of clinical remission	Not applicable
	Maintained for more than 24 months	

Legend: OCS—oral corticosteroids; FeNO—Fractional Exhaled Nitric Oxide.

**Table 3:** Key gaps and challenges in achieving asthma remission.

Criteria	Gaps	Challenges
Multi-factorial	Comorbidities	Their Impact on remission is underexplored data Limited understanding of how eosinophilic phenotype impacts remission and biological efficacy Need for more research on how atopy interacts with asthma remission and biologic treatment
	Eosinophilic	
	Atopic	
Time of intervention	Mild Moderate Severe	No standardized timing for initiating biologics, especially for moderate-to-severe asthma Uncertainty about biologic use across asthma severities
Remission criteria	Symptom control	Maintenance of symptom control remains challenging, particularly in severe asthma
	Reduced exacerbations	Exceptional exacerbation rates persist despite biologic therapy
	Patient and health care provider agreement	Mismatches between patient and clinician expectations regarding remission
Biomarkers	TSLP levels	Need additional evidence to support TSLP as an indicator of remission
	Eosinophils FeNO	Eosinophil-mediated asthma reactions to biologics are heterogeneous and merit additional investigation The role of FeNO in remission and biologic therapy needs further clarification

Legend: TSLP—thymic stromal lymphopoietin; FeNO—Fractional Exhaled Nitric Oxide.

## Data Availability

This review summarizes data reported in the literature.
